# Intradermal Immunoglobulin E (IgE) Injection in a Specific Interval With Emollient in the Control of Chronic Idiopathic Urticaria and Allergic Rhinitis

**DOI:** 10.7759/cureus.48358

**Published:** 2023-11-06

**Authors:** Farhana Rashid Sumi, Md. Abul Kalam A Bhuiyan, Md. Abdul Mukit, Sarker Mahbub A Shamim, Rehnuma Tabassum Orthy, Saimun Jesmin

**Affiliations:** 1 Department of Dermatology, City Medical College and Hospital, Gazipur, BGD; 2 Department of Otolaryngology, City Medical College and Hospital, Gazipur, BGD; 3 Department of Dermatology and Venereology, MH Samorita Hospital and Medical College, Dhaka, BGD; 4 Department of Dermatology and Venereology, Dhaka Dermatology Institute, Dhaka, BGD

**Keywords:** chronic idiopathic urticaria, allergic rhinitis, intradermal ige injection, emollient, dermatology

## Abstract

Background and objective: Urticaria is distinguished by the activation of mast cells and basophils via degranulation, predominantly induced by the cross-linkage of allergens with specific immunoglobulin E (IgE) antibodies. Several hypotheses propose that intradermal injections of IgE stimulate the production of antibodies that are specifically targeted towards the histamine/immunoglobulin complex. Subsequently, these antibodies exhibit binding affinity towards and exert inhibitory effects on the generation of histamine during the occurrence of allergic responses. The administration of many histaglobulin injections results in an increase in the concentration of these specific antibodies. Consequently, the present study was devised to assess the effectiveness of intradermal IgE injection in conjunction with an emollient for managing chronic idiopathic urticaria and allergic rhinitis at varying time intervals.

Methods: This study employed a cross-sectional design and included a sample of 104 participants. The sample was divided into two groups: persons diagnosed with chronic idiopathic urticaria (n=54) and individuals diagnosed with both allergic rhinitis and chronic urticaria (n=50). A 1 ml intradermal IgE injection was provided on a weekly basis over a duration of six months. A total of 49 patients were treated with intradermal IgE injection alone, while 93 patients were treated with intradermal IgE injection combined with emollient application. The evaluation of the treatment's efficacy involved the utilization of the urticaria activity score (UAS) for chronic urticaria, as well as the assessment of symptomatic improvement in cases of allergic rhinitis. The weekly examination was conducted over a span of three consecutive weeks, followed by subsequent evaluations after four, 12, and 24 weeks of attendance, culminating in the final assessment.

Results: Within the sample of 104 participants, a substantial majority of 93 individuals exhibited good outcomes in the management of their condition with the utilization of emollients, whereas a minority of 11 patients experienced inadequate control. In contrast, a group of 49 participants had a therapy regimen that did not include the application of emollients, while another group of 55 persons displayed symptoms that were not effectively managed. Based on recent research findings, a noticeable decrease in symptoms of modest magnitude was observed by the end of the third month. Furthermore, it is important to note that all symptoms were successfully alleviated during a six-month therapeutic regimen, but there was a minor residual dissatisfaction with the impairment of the sense of smell.

Conclusion: Following the administration of six intradermal IgE injections, a significant improvement in symptoms was observed in over 97% of patients, regardless of whether their IgE levels remained unaltered, decreased, or rose.

## Introduction

Urticaria is a frequently seen dermatological disorder distinguished by the manifestation of wheals, angioedema, or a combination thereof [1,2]. The hives are transient, lasting for a short duration ranging from minutes to hours, and resolve without any concurrent secondary cutaneous manifestations, such as scales or excoriations. Pruritus is a prominent distinguishing feature utilized in the process of differential diagnosis. In contrast to wheals, angioedema is distinguished by edematous swellings occurring in the deeper layers of the dermis, subcutis, or submucosa. Compared to wheals, the duration for resolution of these lesions is considerably prolonged, with the potential to linger for up to three days.

It is important to emphasize that identifying these lesions is mainly linked to the sensation of pain rather than itching [1]. The mediators that are released by mast cells generate a localized response that is marked by vasodilation, increased permeability of capillaries, and plasma leakage. This reaction ultimately leads to elevated, red wheals [2]. The stimulation of sensory skin nerves has been identified as the underlying cause for the appearance of itch and the presence of an erythematous halo, which is a direct outcome of the axon reflex [1,2]. Nevertheless, the precise pathophysiological mechanisms responsible for these events have not been wholly understood as of now. The manifestation of wheals and angioedema can occur spontaneously or be triggered by external stimuli, such as exposure to cold water or vertical pressure [3]. Chronic urticaria can be differentiated from acute urticaria based on the persistence of wheals and angioedema for more than six weeks. According to the established international classification, chronic urticaria can be classified into two primary categories: chronic spontaneous urticaria (CSU), which manifests without a discernible cause, and chronic inducible urticaria (CINDU), which is characterized by symptoms that consistently arise due to specific and well-defined circumstances [1].

Urticaria is distinguished by the activation of mast cells and basophils via degranulation, predominantly induced by the cross-linkage of allergens with specific immunoglobulin E (IgE) antibodies [4]. The IgE antibodies are bound to the high-affinity IgE receptor (FcԑRI) situated on the cellular membrane of mast cells and basophils. Novel biological treatments have been developed to selectively target IgE or the high-affinity IgE receptor (FcԑRI) to attenuate mast cell and basophil activation by interrupting this pathogenic pathway [5,6]. Several possibilities suggest that the administration of intradermal IgE injections facilitates the production of antibodies that specifically target the histamine/immunoglobulin complex. Subsequently, these antibodies exhibit affinity for and impede the generation of histamine during instances of allergic responses. The administration of many doses of histaglobulin injection has been found to increase the concentration of these antibodies [7,8]. Hence, the present study assessed the effectiveness of intradermal IgE infusion in conjunction with an emollient for managing chronic idiopathic urticaria and allergic rhinitis at varying time intervals.

## Materials and methods

This cross-sectional study was conducted in the departments of dermatology, venereology, and otolaryngology in City Medical College and Hospital, Gazipur, Bangladesh, from January 2022 to March 2023 after obtaining ethical approval from the same institution (EC number: IEC/NMCRC/APPROVAL/03/2021).

Selection of patients

The study's inclusion criteria encompassed individuals aged 5-56, of any gender, who had been diagnosed with chronic idiopathic urticaria and allergic rhinitis. Additionally, participants were required to be actively using antihistamines as a treatment for both illnesses. Individuals who had previously encountered an unfavorable response after administering IgE injections were excluded. The study did not include women who were pregnant or breastfeeding.

Investigations

Before conducting baseline assessments, patients were provided with information regarding the study's aims and were requested to grant written consent. The researchers recorded demographic variables for each patient, such as age, gender, and weight. Additionally, they evaluated clinical data, such as the length of signs and symptoms, as well as the patient's medical history. After the aforementioned event, the patient received a comprehensive medical assessment. Several diagnostic tests were conducted, including a thorough blood analysis, specifically focusing on the total circulating eosinophil count (TCE). Additionally, the serum IgE level was measured, and an X-ray of the paranasal sinuses (occipitomental (OM) view) was performed.

Investigational procedure

In addition to the ongoing treatment of patients, they received three intradermal administrations of a 1 ml IgE vaccine (HISTOGLOB®, Bharat Serums and Vaccines Limited, Maharashtra, India). This vaccine comprises normal human immunoglobulin (12 mg) and histamine dihydrochloride (0.15 mcg). The initial three weeks of injections were administered every week, while the subsequent injections were administered at intervals of four, 12, and 24 weeks.

Observation and follow-up

Each participant involved in the experiment was obligated to adhere to the designated 24-week duration of the study. At present, a series of intradermal IgE injections were provided over three weeks. Subsequently, follow-up consultations were scheduled for weeks 4, 12, and 24. The disease severity at each visit was evaluated using the urticaria activity score (UAS), a scoring system that is standardized, dependable, and user-friendly. The investigation also examined the mitigation of symptoms associated with allergic rhinitis. The evaluation of the product's safety was conducted by vigilantly monitoring for any occurrences of adverse events (AEs). During each appointment, detailed documentation was made regarding the medications administered simultaneously.

Statistical measurements

The data analysis was conducted using SAS®, Version 9.4 (SAS Institute, Cary, North Carolina, United States). The descriptive statistics for continuous variables were analyzed, including the mean, standard deviation, median, minimum, and maximum values. The study examined the frequency and proportion of categorical variables. The efficacy analysis consisted of all individuals who completed the trial visit on day 28. The Wilcoxon signed-rank test assessed the UAS measurements before treatment (day 0) and after treatment (day 28). A p-value less than 0.05 was considered significant.

## Results

A total of 150 patients were chosen for the intervention. A total of 104 patients successfully finished the full course of treatment. Out of the whole sample, a subset of 50 individuals were diagnosed with chronic idiopathic urticaria, while another subset of 54 individuals were diagnosed with both allergic rhinitis and chronic idiopathic urticaria concurrently. Table 1 presents the demographic characteristics of the participants.

**Table 1:** Baseline demographic data of the participants N: total number; mean±SD: mean and standard deviation

Characteristics	Parameters N/mean±SD
Mean age (years)	28.98±2.8
Female	75 (72.11%)
Male	29 (27.8%)
Urticaria with angioedema	21 (20.1%)
Urticaria without angioedema	83 (79.8%)
Mean duration (months)	6.8±6.4

**Figure 1:**
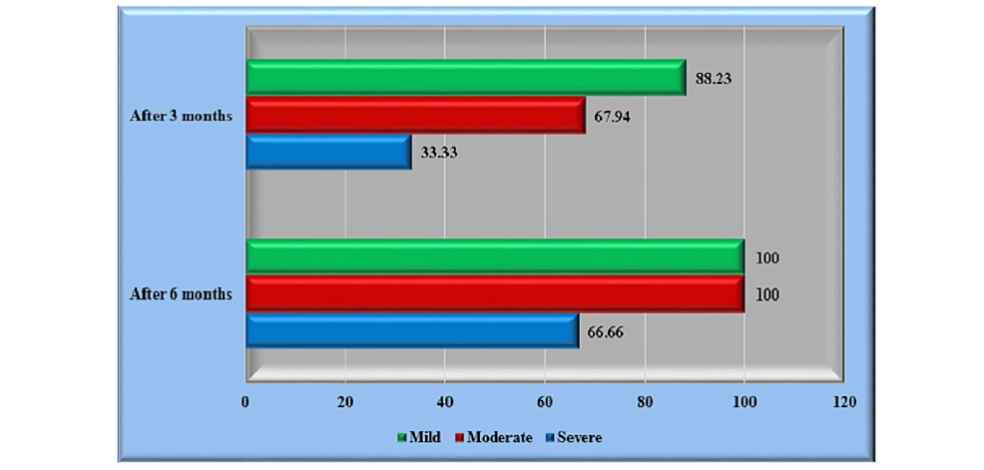
The reduction in number and UAS three and six months later UAS: urticaria activity score Mild (0-6), moderate (7-12), and severe (13-18)

A decline in the number of UAS was seen on a monthly basis. Figure 1 presents the first emergence of the reaction, which became evident during the fourth week and had substantial growth over the third and sixth months. Based on the findings of the UAS, 17 patients had mild symptoms; among these patients, two individuals (1.92%) did not experience any improvement in their condition after three months. However, it is noteworthy that all patients in the mild group had complete recovery from their sickness by the sixth month of the study (p<0.0001). Meanwhile, 78 patients reported moderate to severe UAS; among them, it was seen that 25 patients (24.03%) did not experience any discernible improvement after the third month. However, it is noteworthy that the subset of patients classified as mild exhibited complete recovery from their ailment by the sixth month (p=0.0002). In contrast, three individuals, accounting for 2.88% of the total patient population, exhibited no discernible response upon the conclusion of the comprehensive therapy regimen (Figure 1). 

**Figure 2:**
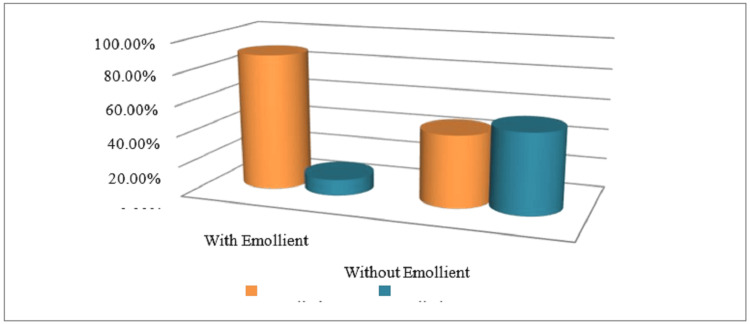
The reduction of patients' symptoms at the baseline and after six months with the use of an emollient (olive oil, lotion, glycerin)

Following the completion of an extensive treatment regimen, the patients exhibited symptoms of allergic rhinitis accompanied by chronic idiopathic urticaria. Notably, there were no observed amelioration in nasal blockage and a decline in olfactory function during the initial month (Figure 2).

Out of a total of 104 patients, a majority of 93 individuals exhibited successful management of their condition through the use of emollients, whereas a minority of 11 patients experienced inadequate control. In contrast, a group of 49 individuals received treatment without the use of emollients, while another group of 55 patients experienced uncontrolled symptoms. The latest research indicates that there was a moderate alleviation of symptoms observed following the completion of the third month. Furthermore, it is noteworthy that all symptoms were successfully alleviated during a six-month course of treatment, but with a minor residual dissatisfaction with olfactory impairment (Table 2).

**Table 2:** Immunological response after the administration of IgE injection in allergic rhinitis IgE: immunoglobulin E; -: no response; +: fair; ++: satisfactory; +++: good; ++++: excellent

Duration	Sneezing	Discharge	Blockage	Impairment of smell
First month	+	+	-	-
Second month	+	+	+	-
Third month	++	++	++	+
Fourth month	+++	+++	++	+
Fifth month	+++	+++	+++	++
Sixth month	++++	++++	+++	++

## Discussion

Chronic urticaria, an idiopathic disorder, exerts a substantial detrimental effect on the overall quality of life of those afflicted over an extended period. The primary therapeutic strategy for chronic urticaria continues to be the use of non-sedating antihistamines [7-12]. While it does offer rapid symptom relief, its impact on the immunopathogenesis or progression of the disease is modest. The lack of a pharmacological strategy that mainly targets the early stages of the immune response and offers long-lasting effects remains a challenge in effectively managing episodes of urticaria. There are several immunological pharmacological drugs available, such as intravenous immunoglobulin, methotrexate, oral corticosteroids, tacrolimus, cyclosporine, and methotrexate. However, the current body of research does not provide substantial evidence for their utilization [8]. The administration of intradermal IgE injection has demonstrated encouraging therapeutic outcomes in many allergy disorders, as evidenced by previous research [13]. The molecule under consideration is a sterile amalgamation of gamma globulin, a physiologically active protein fraction obtained from human blood, and histamine dihydrochloride. Based on the concept, it is hypothesized that administering IgE injection therapy can decrease IgE levels by augmenting the patient's capacity for serum histamine binding [14]. The study sample comprised 104 patients who had been diagnosed with chronic idiopathic urticaria and allergic rhinitis. Out of the total number of participants, 29 were identified as male, while 75 were identified as female. The mean age of the participants was 34 years, accompanied by a standard deviation of eight years. The average score for the reduction of urticaria demonstrated a statistically significant decrease over three months. After 24 weeks of medication, it was shown that individuals with mild to moderate cases of urticaria experienced an improvement in their UAS.

The investigation conducted by Rajesh et al. revealed that a majority of patients exhibited favorable outcomes, which aligns with the results obtained in our study [15]. A longitudinal study was born with a sample size of 2000 persons to investigate the occurrence of adverse responses linked to intradermal IgE injections. The study's findings indicated that 0.5% of the patients had these undesirable side effects. The documented detrimental outcomes encompass symptoms such as lethargy, malaise, headaches, and localized reactions [14]. The results of this investigation also demonstrated that the administration of intradermal IgE injection is generally considered to be safe and well-tolerated, as supported by the occurrence of only one case of localized redness at the injection site in a solitary participant. Based on the results of our research, it was observed that a treatment period of 12 weeks resulted in a symptomatic enhancement above 50% for patients diagnosed with allergic rhinitis and urticaria.

In addition, the complete treatment regimen significantly reduced symptoms, as seen by 81.25% of subjects reporting relief. This observation aligns with the research undertaken by Gushchin et al. [16]. The study's findings indicate that a considerable percentage of individuals, particularly 89.42%, reported having relief from chronic idiopathic urticaria when subjected to a treatment regimen involving a combination of IgE injection and emollient. On the other hand, the administration of intradermal IgE injection as an independent therapeutic intervention yields a positive effect in 47.11% of patients diagnosed with chronic idiopathic urticaria. Despite doing exhaustive searches on reputable academic databases such as ScienceDirect, Google Scholar, and PubMed, our efforts to locate studies that successfully merged both modalities yielded no results. The present analysis highlights the significance of the findings by illustrating the correlation between regular intradermal IgE injections combined with emollients and prior investigations conducted in diverse geographical settings.

Our investigation is subject to certain inherent constraints. The primary limitation of this study was associated with the chosen research methodology and the restricted size of the sample population. The cross-sectional research approach is limited in proving causation due to its inherent non-randomized nature and the challenge of determining the directionality of associated variables. Furthermore, it is worth noting that the study lacked a control or placebo group, which would have allowed for meaningful comparisons.

## Conclusions

Individuals suffering from chronic idiopathic urticaria and allergic rhinitis have more significant relief after therapy with intradermal IgE injections with emollient application and benefit from a longer period of remission without requiring additional therapeutic measures. The administration of this injection did not result in any adverse effects. Additionally, a significant level of pain relief was recorded by patients following the administration of intradermal IgE therapy combined with emollient application. Further investigation should be conducted using a significant sample size in order to evaluate its efficacy.
